# Avian Mite Dermatitis Caused by *Microlichus sp.* in the Great Kiskadee *(Pitangus sulphuratus)*

**DOI:** 10.1007/s11686-026-01226-z

**Published:** 2026-03-02

**Authors:** Fabiane de Holleben Camozzato Fadrique, Thais Fernanda de Jesus, Filipe Obelar Martins, Eduarda Saldanha Rieffel, Maria Lucia Rösler, Lucas Almeida de Souza, Camila Belmonte Oliveira, Raqueli Teresinha França

**Affiliations:** 1 Department of Veterinary Clinics, Federal University of Pelotas (UFPel), Pelotas, Rio Grande do Sul Brazil; 2 Núcleo de Reabilitação da Fauna Silvestre (NURFS), Federal Universityof Pelotas (UFPel), Pelotas, Rio Grande do Sul Brazil; 3 Laboratório de Protozoologia e Entomologia (LAPEN), Department ofMicrobiology and Parasitology, Federal University of Pelotas (UFPel), Pelotas, Rio Grande do Sul Brazil

**Keywords:** Cutaneous acariasis, Epidermoptidae, Parasitic skin disease, Passerine hosts, Wildlife clinical management

## Abstract

**Purpose:**

To report a case of avian dermatitis associated with *Microlichus sp.* (Acari: Epidermoptidae) in a free-ranging *Pitangus sulphuratus* (Great Kiskadee) from southern Brazil, emphasizing the clinical presentation and parasitological diagnosis.

**Methods:**

A juvenile *P. sulphuratus* was rescued and admitted to a wildlife rehabilitation center presenting feather loss and cutaneous lesions. Crust samples were collected from affected areas and examined microscopically after clarification in lactophenol. Mites were identified morphologically using classical and contemporary taxonomic keys. Topical ivermectin (0.4 mg/kg) was administered once daily for 10 consecutive days, and clinical evolution was monitored during rehabilitation.

**Results:**

Numerous mites morphologically consistent with *Microlichus* sp. were observed, supporting the diagnosis of epidermoptid infestation. Progressive resolution of dermatological lesions and complete feather regrowth were observed following treatment; however, no post-treatment parasitological reassessment was performed. To our knowledge, this represents the first clinical report of *Microlichus* sp. associated with dermatitis in *P. sulphuratus* in Brazil.

**Conclusion:**

This case highlights the relevance of integrating clinical and parasitological investigations in wildlife rehabilitation settings and contributes to expanding current knowledge on the host range and potential health impacts of epidermoptid mites in free-ranging Neotropical birds.

## Introduction

The Great Kiskadee (*Pitangus sulphuratus*) is a widespread Neotropical passerine (Tyrannidae) listed as Least Concern by the IUCN Red List. The species is classified as Least Concern due to its extremely large geographic distribution and increasing global population trend. According to the IUCN, its native range extends from the southern United States (Texas) through Mexico and Central America to most of South America, including Brazil, Argentina, Paraguay, Uruguay, Bolivia, Peru, Colombia, Venezuela, and the Guianas. This broad distribution is associated with high ecological adaptability, as the species occurs in a wide variety of habitats, including subtropical and tropical forests, mangroves, shrublands, grasslands, agricultural landscapes, and urban areas. Such ecological flexibility increases the likelihood of exposure to diverse environmental pathogens and ectoparasites, reinforcing the relevance of documenting parasitic conditions in this species [1].

Birds serve as hosts for mites that can affect both their integumentary and respiratory systems [2–4]. Among these, members of the family Epidermoptidae are specialized skin mites that may proliferate on the epithelial surface and within feather follicles [2, 5]. The genus *Microlichus* includes highly specialized ectoparasites, primarily reported in passerine birds, where they are associated with intense desquamation [6, 7]. Clinical signs associated with infestations by *Microlichus* sp. may include feather loss, dermatitis, hyperplasia, hyperkeratosis and pruritus [6, 8].

*Microlichus* spp. have previously been reported in other bird species, such as the saffron finch (*Sicalis flaveola*) and the northern bobwhite (*Colinus virginianus*) [8, 9], reinforcing the ability of this genus to parasitize diverse avian hosts. Lindholm, Venter and Ueckermann [10] identified *M. americanus* in the southern red bishop (*Euplectes orix*) and the spectacled weaver (*Ploceus ocularis*), while Brown and Wilson [11] recorded *M. avus* in a house sparrow (*Passer domesticus*) without apparent clinical signs. These reports indicate that infestation by *Microlichus* does not invariably result in overt disease and that clinical expression may vary according to host-related and environmental factors.

Brazil is one of the most biodiverse countries in terms of avifauna, yet knowledge regarding its acarofauna remains limited [4, 12]. Recent taxonomic surveys conducted in Brazil and neighboring Neotropical regions have revealed a continuous expansion of avian mite diversity, including members of the family Epidermoptidae, emphasizing the need for updated morphological approaches when diagnosing mite infestations in wild birds [13, 14].

In this context, the objective of this study is to report the occurrence of parasitism by *Microlichus* sp. in a free-ranging *Pitangus sulphuratus* presenting clinical signs of dermatitis in the state of Rio Grande do Sul, Brazil.

## Materials and Methods

A juvenile *Pitangus sulphuratus* of undetermined sex and a body condition score of 3/5 was admitted to the Wildlife Rehabilitation Center and Screening Center for Wild Animals of the Federal University of Pelotas (NURFS-CETAS/UFPel), located in Rio Grande do Sul, Brazil. Age classification was based on a combination of criteria commonly used in free-ranging passerines, including plumage characteristics, degree of cranial ossification, body mass, overall body size, feather development (particularly the contour and remiges), and the appearance of the rictal flanges, which are typically more prominent and pale in juveniles. The free-ranging individual was housed in an individual enclosure and received routine supportive care.

Crust samples from the affected area were collected and placed in sterile microtubes. Because the material was delivered immediately to the Laboratory of Protozoology and Entomology (LAPEN) within the same institution, no preservative was used and the samples were kept at room temperature until processing.

The material was cleared in Aman’s lactophenol and mounted on glass slides following standard acarological techniques for the preparation of parasitic mites, allowing clear visualization of diagnostic morphological structures. Microscopic examination was performed under a Zeiss V20¹ light microscope with a 10× eyepiece, using 4×, 10×, and 40× objectives, (resulting in total magnifications of 40×, 100× and 400×). Mite identification was conducted using the identification keys and protocols described by Furman and Tarshis [15] and Fain [16].

To strengthen morphological accuracy, modern diagnostic criteria from recent Epidermoptidae systematics were also incorporated, including details of peritremal curvature, dorsal shield sculpturing, posterior opisthosomal lobe morphology, tarsal chaetotaxy, and sexual dimorphism patterns described in contemporary studies [13, 14, 17]. These additional frameworks were essential to reliably distinguish *Microlichus* from morphologically similar genera known to occur in Brazil.

Topical ivermectin (0.4 mg/kg) was administered once daily for 10 consecutive days. The drug was applied directly to the affected cranial region, covering areas of visible crusting and feather loss, with care taken to avoid contact with the eyes and nares. Treatment was restricted to the dorsal cranial surface rather than the entire body. Histopathological examination was not performed.

## Results

Upon admission, the bird presented focal feather loss in the crown region, considered abnormal due to its localized distribution, and mild crusting of the rhamphotheca, which was recorded as a nonspecific finding.

**Fig. 1:**
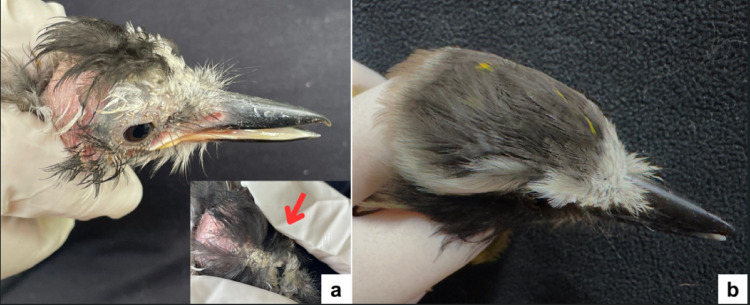
*Pitangus sulphuratus*. **A** Dermatological alterations associated with mite infestation: rostral region showing localized feather loss; Inset: whitish crusts on the crown of the head (arrow). **B** Complete feather regrowth in the previously affected area observed at discharge after 60 days of rehabilitation following topical ivermectin treatment

During hospitalization, the animal exhibited marked progression of feather loss on the head, accompanied by increased epidermal desquamation, whitish crust formation, and hyperkeratosis in the affected area (Fig. 1 - A). By the end of the treatment period, complete feather regrowth was observed in the previously affected regions (Fig. 1B).

The bird was transferred to the rehabilitation program and released back into the wild after 60 days, with no recurrence of clinical signs observed during captivity.

**Fig. 2:**
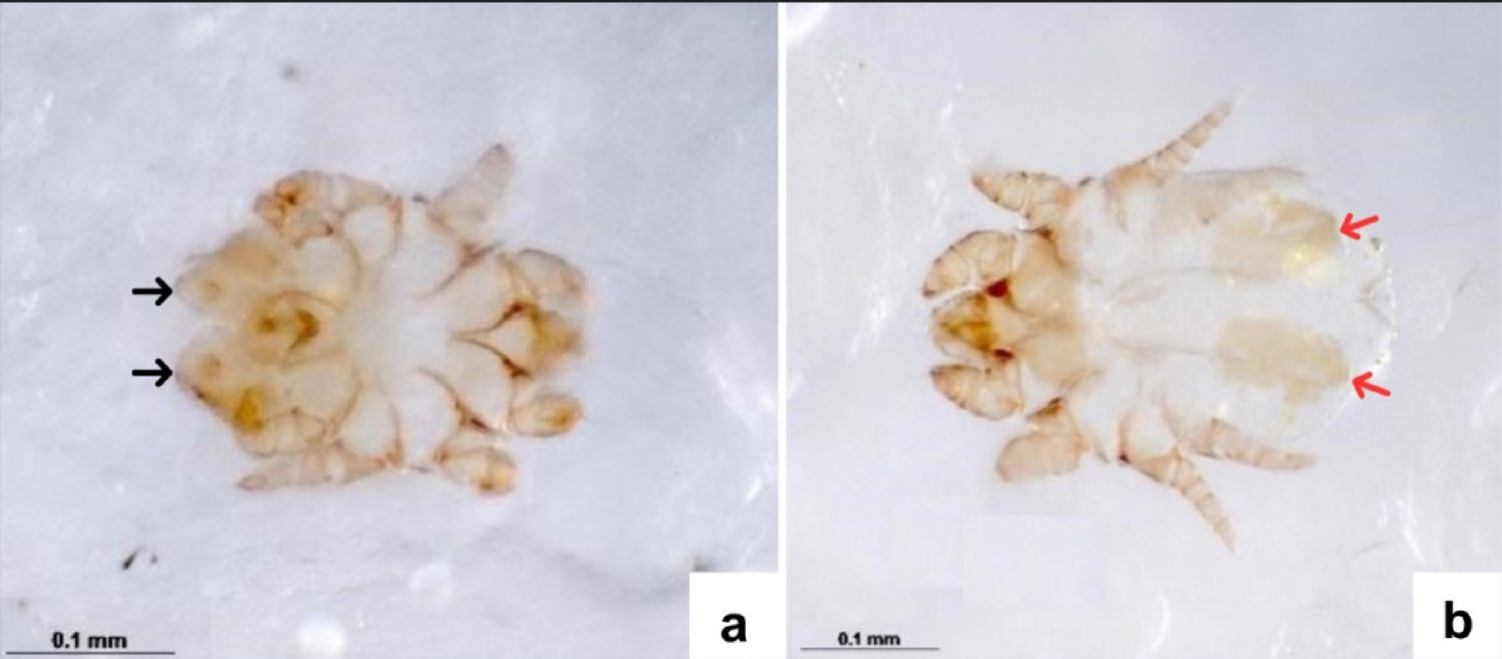
*Microlichus* sp. in dorsal view under light microscopy. **A** Male mite showing bilobed posterior copulatory suckers (black arrow), short and robust legs, and a compact, oval body shape. **B** Female mite with a rounded posterior body region, presence of a hysteronotal plate (red arrow), and a single claw on tarsus II, diagnostic features consistent with the genus *Microlichus* (Epidermoptidae)

The microscopic examination of the prepared slides revealed numerous mites with morphological characteristics consistent with the genus *Microlichus* (Order Sarcoptiformes, Family Epidermoptidae) (Fig. 2), classified according to dichotomous keys [6, 7]. The analyzed specimens exhibited body lengths ranging from 0.17 to 0.39 mm, short and robust legs, an oval and relatively flattened body, and rounded peritremes, which are general features shared by several epidermoptid genera. Genus-level identification was therefore not based solely on these traits, but on the presence of additional diagnostic characters observed in cleared specimens. In these specimens, both male and female mites exhibited well-defined characteristics consistent with the genus *Microlichus*. Males showed bilobed posterior copulatory suckers, whereas females presented hysteronotal plates and a single claw on tarsus II, in addition to a crescent-shaped chitinous band posterior to the anus on both.

To ensure correct genus identification, specimens were evaluated based on a combination of diagnostic characters traditionally considered essential for the genus *Microlichus* within the family Epidermoptidae. Closely related genera were excluded based on the absence of key morphological traits, including the elongated opisthosomal lobes and modified tibial setae characteristic of *Myialges*, as well as the elongated stylophore and distinctive gnathosomal morphology described for *Epidermoptes*. Additionally, specimens did not exhibit the posterior opisthosomal configuration reported for other epidermoptid genera [6, 8, 15, 16]. The combined presence of male and female diagnostic characters, together with the exclusion of morphologically similar genera, was consistent with published descriptions of the genus *Microlichus* and allowed reliable differentiation from other epidermoptid mites.

## Discussion

Parasitism by *Microlichus* sp. was associated with a clinical presentation of avian dermatitis in this case, characterized by feather loss, pityriasis, and hyperkeratosis, with clear progression during hospitalization and subsequent improvement following treatment. Similar clinical manifestations have been reported in association with infestations by mites of this family, although the presence of infestation does not invariably result in overt disease and clinical expression may be variable among hosts [2, 7, 8, 10, 18–21]. Epidermal desquamation is considered to be related to the feeding activity of these mites [7–9, 22], which is consistent with the marked increase in scaling and crust formation observed in the affected areas of the present bird. The absence of clinical signs in a house sparrow infested by *Microlichus avus* [11] further highlights the variable clinical expression associated with these ectoparasites.

Mites of the genus *Microlichus* are primarily associated with birds, although occasional associations with mammals have been reported. They typically inhabit superficial epidermal layers, feeding on skin debris and secretions, a pattern compatible with the superficial and localized dermatological lesions documented in this case [7, 22].

In birds, the integument is characterized by a relatively thin epidermis, reduced adnexal structures compared to mammals, and extensive dermal vascularization, particularly in feathered regions, features that may influence the cutaneous disposition of lipophilic compounds applied topically [23]. In addition, the high density of feather follicles and associated lipid-rich secretions may favor local retention on the integumentary surface, supporting the rationale for topical drug delivery in avian dermatological conditions, particularly when lesions are superficial and localized, as observed in this case [23, 24].

Recent studies conducted in Brazil and other Neotropical regions have expanded knowledge of avian mite fauna through new records and host associations, highlighting the still limited understanding of mite diversity in wild birds [13, 14]. In this context, the present report adds clinical evidence to a body of literature that remains largely taxonomic, as most regional contributions have focused on feather-associated mites. The detailed morphological evaluation performed herein was therefore essential to support genus-level identification in a clinical setting, particularly among groups with overlapping morphological traits. Ecological investigations indicate that environmental conditions and reproductive cycles may influence mite prevalence [25], which may help explain the occurrence and progression of infestation in this free-ranging individual.

Although the route of infestation could not be determined, transmission of these mites is thought to occur through direct contact between infected and non-infected hosts or via contaminated environments such as nests and resting sites [17]. Phoretic association with flies (Diptera: Hippoboscidae), which inhabit nests and individual birds, represents another plausible route of transmission, as these insects are known vectors of various organisms, including arthropods [3, 17, 19, 26]. Additionally, the omnivorous feeding habits of *Pitangus sulphuratus*, including predation on insects, mollusks, nest contents, and small mammals such as bats [27], may increase exposure to infested prey or contaminated environments, as suggested for other avian hosts [2, 3].

Topical ivermectin was associated with progressive clinical improvement in this case, including cessation of feather loss and complete regrowth in previously affected areas, supporting a temporal association between treatment and clinical recovery. Nevertheless, true therapeutic efficacy cannot be confirmed, as post-treatment skin scrapings were not performed to document mite elimination. Previous studies have reported variable responses to ivermectin treatment in epidermoptid infestations, including lack of clinical remission following oral protocols with extended intervals [8], reinforcing that the outcome observed here should be interpreted as a clinical response rather than confirmation of parasitological cure. From a clinical standpoint, therapeutic approaches for ectoparasitic mite infestations do not differ substantially among Astigmata, Mesostigmata, or other mite groups causing superficial dermatological disease in birds. Accordingly, the use of topical ivermectin has been widely reported across different avian acariasis, including epidermoptid infestations, following a comparable clinical rationale rather than taxon-specific protocols [17].

The use of topical ivermectin was guided by practical and physiological considerations. Topical application allows direct exposure of mites inhabiting superficial epidermal layers to a lipophilic macrocyclic lactone. This approach is consistent with the feeding behavior and microhabitat of epidermoptid mites [7, 22]. In addition, topical treatment minimizes handling time and stress in free-ranging passerines and avoids variability associated with oral intake, which may be compromised in debilitated or anorectic birds [28]. Clinical reports support the effectiveness of topical or spray ivermectin in avian mite infestations, including pigeons and spot-on applications in small passerines and psittacines, with reduction of lesions and/or mite burden [24, 28, 29].

Ivermectin exerts its acaricidal effect by binding to glutamate-gated chloride channels in arthropods, leading to neuromuscular paralysis and death. Experimental studies have demonstrated potent acaricidal activity against avian mites, including *Dermanyssus gallinae*, supporting the biological plausibility of the clinical improvement observed following topical application, although these findings derive primarily from experimental exposure models [30].

Despite these encouraging outcomes, the pharmacological basis for ivermectin’s distribution and efficacy in birds must be interpreted cautiously. Pharmacokinetic data derived from mammals cannot be directly extrapolated to avian species, and studies evaluating topical absorption and tissue distribution in birds remain limited. Proposed mechanisms related to lipophilicity and host–parasite interactions have been suggested. However, these mechanisms have not yet been fully demonstrated in avian species [31–34]. Poultry data indicate rapid systemic distribution and elimination following oral administration, with peak plasma concentrations occurring within hours and declining thereafter [35]. Therefore, while the clinical evolution observed here is compatible with a therapeutic effect, definitive conclusions should be drawn cautiously, and controlled pharmacokinetic and parasitological studies are needed to establish optimal treatment protocols in wild birds.

Finally, despite the success of morphological identification, the lack of molecular confirmation represents an important limitation, restricting taxonomic resolution to the genus level. As this report describes a single clinical case, broader ecological or epidemiological inferences cannot be made. Histopathological examination was also not performed because the lesions were superficial and showed progressive clinical improvement during treatment, suggesting that invasive diagnostic procedures would be unlikely to provide additional clinically relevant information in this context.

## Conclusion

The identification of *Microlichus* sp. infesting *Pitangus sulphuratus* broadens the known host spectrum of this genus and contributes to a more comprehensive understanding of the diversity and pathogenic potential of epidermoptid mites in Neotropical passerines. This case reinforces the importance of integrating clinical and parasitological approaches in wildlife rehabilitation settings to support early detection of ectoparasitic diseases and inform broader surveillance of avian health.

## Data Availability

No datasets were generated or analysed during the current study.
